# gC1qR/C1qBP/HABP-1: Structural Analysis of the Trimeric Core Region, Interactions With a Novel Panel of Monoclonal Antibodies, and Their Influence on Binding to FXII

**DOI:** 10.3389/fimmu.2022.887742

**Published:** 2022-07-05

**Authors:** Ying Zhang, Alexander J. Vontz, Ethan M. Kallenberger, Xin Xu, Nicoleta T. Ploscariu, Kasra X. Ramyar, Brandon L. Garcia, Berhane Ghebrehiwet, Brian V. Geisbrecht

**Affiliations:** ^1^ Department of Biochemistry & Molecular Biophysics, Kansas State University, Manhattan, KS, United States; ^2^ Department of Medicine, Stony Brook University, Stony Brook, NY, United States

**Keywords:** gC1qR/C1qBP/HABP-1, FXII, protein-protein interactions, monoclonal antibodies, inhibitors

## Abstract

The protein gC1qR/C1qBP/HABP-1 plays an essential role in mitochondrial biogenesis, but becomes localized at the cellular surface in numerous pathophysiological states. When this occurs on endothelial cells, surface-exposed gC1qR activates the classical pathway of complement. It also promotes assembly of a multi-protein complex comprised of coagulation factor XII (FXII), pre-kallikrein (PK), and high-molecular weight kininogen (HMWK) that activates the contact system and the kinin-generating system. Since surface-exposed gC1qR triggers intravascular inflammatory pathways, there is interest in identifying molecules that block gC1qR function. Here we further that objective by reporting the outcome of a structure/function investigation of gC1qR, its interactions with FXII, and the impact of a panel of monoclonal anti-gC1qR antibodies on FXII binding to gC1qR. Although deletion mutants have been used extensively to assess gC1qR function, none of these proteins have been characterized structurally. To that end, we determined a 2.2 Å resolution crystal structure of a gC1qR mutant lacking both of its acidic loops, but which retained nanomolar-affinity binding to FXII and FXIIa. This structure revealed that the trimeric gC1qR assembly was maintained despite loss of roughly thirty residues. Characterization of a novel panel of anti-gC1qR monoclonal antibodies identified several with biochemical properties distinct from previously described antibodies, as well as one which bound to the first acidic loop of gC1qR. Intriguingly, we found that each of these antibodies could partly inhibit binding of FXII and FXIIa to gC1qR. Based on these results and previously published studies, we offer new perspectives for developing gC1qR inhibitors.

## Introduction

gC1qR/C1qBP/HABP-1 is a multicompartmental and multifunctional protein that undergoes spatiotemporal changes in its distribution in response to various cellular and systemic stimuli (1). Although it normally functions within the cell and plays an essential role in mitochondrial biogenesis by virtue of its mRNA binding activity (2), gC1qR exposed at the cellular surface serves as a route to gain cellular entry for numerous pathogens as well as acting as an inflammatory trigger in a number of other diseases including vascular disorders and various cancers [Reviewed in (1)]. Because of its multicompartmental distribution and lack of a transmembrane domain, gC1qR does not meet the criteria of a classical receptor. Instead, it is considered as a prototype of a ‘danger associated’ or ‘damage associated’ molecular pattern by virtue of its enhanced surface expression during infection or inflammatory processes. Consequently, its recognition by components of the innate immune system can initiate potent inflammatory reactions that promote disease, including the complement system, the contact pathway, and the kinin-generating system (1).

Histological studies have shown that gC1qR is expressed on epithelial tumors of diverse origins (3) and elevated levels of gC1qR have likewise been correlated with poor clinical outcomes in breast cancer patients (4, 5). Although these relationships have not been defined as causal, a more contemporary study has shown that anti-gC1qR therapy slows tumor growth in an animal model of breast cancer (6). This emerging proof-of-concept evidence suggests that therapeutic targeting of cell surface-exposed gC1qR may be beneficial in certain cancers. Naturally, it raises questions of whether an analogous approach could be applicable to other diseases as well. A recent report suggests that anti-gC1qR therapy merits further investigation for treatment of specific types of angioedema (7), although this line of inquiry would be strengthened by additional studies using animal models of disease. Nevertheless, these observations have collectively rekindled interest in understanding how gC1qR interacts with various ligands to promote extracellular inflammatory reactions, and how these are influenced by the structure of gC1qR.

The gC1qR transcript encodes a pre-pro protein of 282 amino acids from which an N-terminal segment (i.e. residues 1-73) is removed by site-specific cleavage (8). The mature form of the gC1qR polypeptide therefore consists of residues 74-282 (1) and its crystal structure has been solved to 2.25 Å resolution (9). This structure showed an unusual fold comprised of two prominent N- and C-terminal α-helices positioned more or less anti-parallel to one another, with a relatively large, seven-stranded anti-parallel β-sheet at its core. In addition to this, a number of other key structural features of the gC1qR protein were also revealed. Chief among these is oligomerization of the gC1qR monomer into a toroidal, ring-like trimer (9). The gC1qR ring has an outer diameter of approximately 75 Å, an inner diameter of roughly 20 Å, and is approximately 30 Å thick throughout its circumference (9). Although the biological implications of gC1qR oligomerization remain unclear, the abundance of acidic residues in its sequence, combined with its unusual fold, imparts an asymmetric charge distribution to the gC1qR trimer. Whereas one aspect of the gC1qR trimer has an overall neutral charge, the opposite face of the trimer is rich in negative charge character (9). This noteworthy feature has influenced subsequent predictions regarding gC1qR interactions with its various ligands (1, 10). The contributions of two loops, comprised of residues 139-163 and 190-201 and which connect strands β3-β4 and β5-β6, respectively, have also been the source of some speculation (10). Although these loops are disordered in the gC1qR crystal structure (9), they themselves are rich in acidic residues and project outward from the negatively charged face of the gC1qR trimer. This has raised questions about the role these two loops play in ligand binding, such as whether they function independently or if they cooperate with residues/sites derived from the core of the gC1qR trimer.

Although many endogenous and exogenous ligands for gC1qR have been described (1), there remains little structural information on how these molecules bind to gC1qR. A significant development in this area occurred recently when Kaira et al. reported a crystal structure of gC1qR bound to the ~10 kDa type-II fibronectin domain that is found near the N-terminus of human FXII (hereafter FXII-FNII) (11). This study identified the second acidic loop of gC1qR as a major binding determinant for FXII-FNII, and provided new insights into assembly of the multiprotein complex between gC1qR, FXII, HMWK, and kallikrein that leads to activation of the contact system as well as the kinin-generating system (11). However, the fragment used in that study represents only a small portion of full-length FXII, suggesting that other important features of the FXII/gC1qR interaction may remain to be discovered. We undertook the current study to investigate that possibility, as well as to further define the structural features of gC1qR that mediate its interactions with FXII and other ligands relevant to initiating the contact pathway. We also describe identification of a new panel of anti-gC1qR monoclonal antibodies and show that these antibodies can partly inhibit FXII binding to gC1qR even though they recognize distinct epitopes on the gC1qR protein. Together, these results further our understanding of gC1qR structure/function and lay the foundation for future development of gC1qR-targeted inhibitors.

## Materials & Methods

### gC1qR Proteins

Samples of wild-type gC1qR were overexpressed and purified from recombinant strains of *E. coli.* Briefly, a designer gene fragment encoding residues 74-282 of human gC1qR was subcloned into the prokaryotic expression vector pT7HMT (12) and the resulting sequence-confirmed plasmid was used to transform competent cells of *E. coli* strain BL21(DE3). Cells were cultured in 1 L of terrific broth using standard procedures, and protein expression was induced overnight at 18°C by addition of IPTG (1 mM final concentration). Unless otherwise indicated, all purification steps were carried out at room temperature. Following cell harvest, the induced cell pellet was resuspended and processed for NiNTA affinity chromatography under native conditions as previously described (13). The polyhistidine tag was removed from the recombinant protein by digestion with TEV protease using a 100:1 mass ratio of target protein to protease and incubating overnight at room temperature (12); following removal of imidazole by buffer exchange, the digested sample was reapplied to an NiNTA affinity column and the unbound fraction was collected. The protein sample was further purified by gel-filtration chromatography using a Superdex S200 26/60 column (Cytiva Life Sciences) that had been previously equilibrated in PBS (pH 7.4). Fractions were analyzed by SDS-PAGE and those containing purified, trimeric gC1qR were pooled, concentrated, quantitated, and stored at either 4 or -80°C for later use.

**Figure 1:**
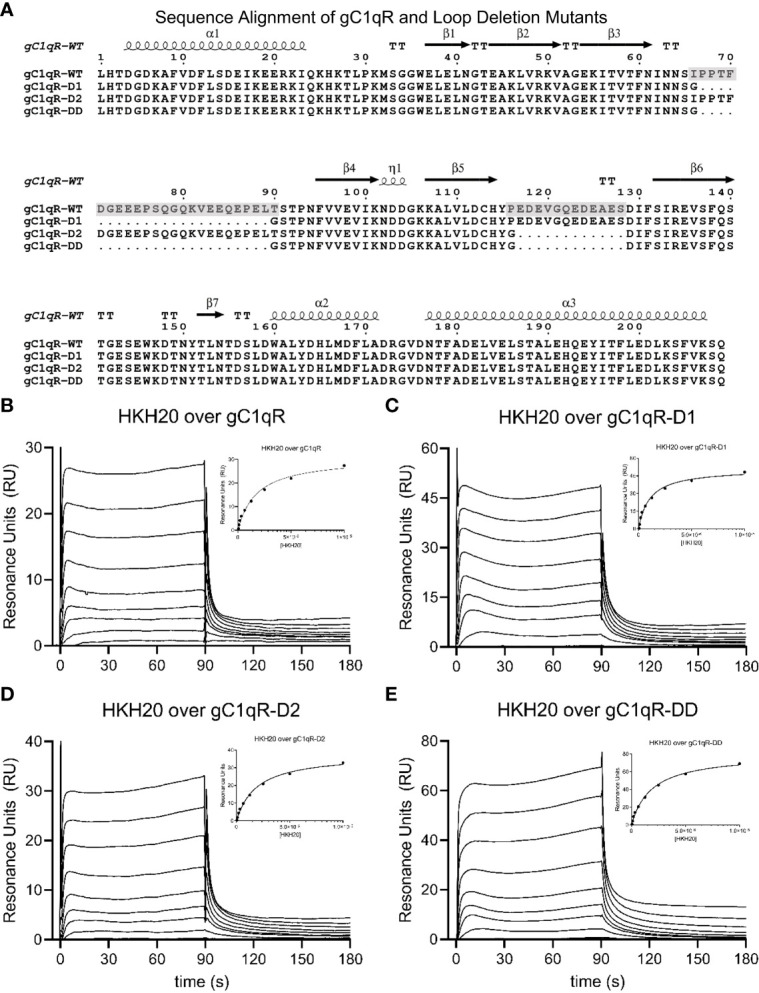
Generation and Characterization of a Panel of Loop-deletion Mutants in gC1qR. **(A)** Sequence/structure alignment of the mature form of wild-type gC1qR and three deletion mutants wherein either one or both negatively charged loops have been replaced by a short glycine linker. The locations of secondary structure elements, as seen in the crystal structure of wild-type gC1qR (9), are shown above the alignment. Transparent grey boxes highlight the locations of the first and second acidic loops in the wild-type gC1qR sequence. Please note that the first residue in this alignment corresponds to Leu 74 in the human gC1qR sequence. **(B)** Surface plasmon resonance characterization of the interaction between wild-type gC1qR and peptide HKH-20 derived from human high-molecular weight kininogen. Reference-corrected sensorgrams for a two-fold dilution series of HKH-20 peptide (10 mM highest concentration) injected over a surface of immobilized gC1qR. A plot where SPR response immediately prior to injection stop is shown as a function of HKH-20 concentration is inset. Data shown in this panel were modified from Ghebrehiwet et al. (1) **(C)** Surface plasmon resonance characterization of the interaction between mutant gC1qR-D1 and peptide HKH-20. **(D)** Surface plasmon resonance characterization of the interaction between mutant gC1qR-D2 and peptide HKH-20. **(E)** Surface plasmon resonance characterization of the interaction between mutant gC1qR-DD and peptide HKH-20.

Gene fragments encoding the gC1qR mutants gC1qR-D1, gC1qR-D2, and gC1qR-DD (**Figure 1A**) were prepared using gene synthesis (GenScript USA). Following sequence confirmation, each fragment was cloned into pT7HMT and used to transform competent cells of *E. coli* strain BL21(DE3). Each mutant protein was overexpressed and purified using a procedure identical to that used for wild-type gC1qR. Gene fragments encoding either gC1qR or gC1qR-DD with a C-terminal avi-tag sequence (i.e. GLNDIFEAQKIEWHE) prior to the stop codon were likewise prepared using gene synthesis, cloned into pT7HMT, and used to transform *E. coli* strain BL21(DE3) cells. As above, expression and purification of gC1qR-avi or gC1qR-DD-avi was accomplished through procedures identical to those used for the wild-type gC1qR protein.

Both chemical and enzymatic protein biotinylation strategies were used in the course of this study. Modification of wild-type gC1qR or gC1qR-DD *via* their surface-exposed amine groups was accomplished using the EZ-link NHS-PEG4-Biotin reagent according to manufacturer’s suggestions (ThermoFisher). Reactions were carried out to yield sub-stoichiometric labeling to avoid modification of all surface-exposed amines. gC1qR-avi and gC1qR-DD-avi were biotinylated *in vitro* using recombinant BirA enzyme and the general strategy reported in a previous publication (14). The biotinylation reaction was carried out in a buffer of 20 mM HEPES (pH 7.4), 140 mM NaCl, 5 mM MgCl_2_ and contained 200 μM target protein, 4 mM ATP, 300 µM biotin, and 2 µM of a BirA fusion protein (14). Successful biotinylation of all proteins was confirmed by blotting and detection with an HRP-streptavidin conjugate according to manufacturer’s suggestions (Thermo Scientific #21130).

### Other Proteins and Peptides

Samples of native (HMWK) and activated High Molecular Weight Kininogen (HMWKa) were obtained from Enzyme Research Laboratories. Peptide HKH-20, which corresponds to the gC1qR-binding motif from human HMWK (sequence HKHGHGHGKHKNKGKKNGKH), was synthesized and purified by GenScript. Samples of native (FXII) and activated Factor XIIa (FXIIa) were also obtained from Enzyme Research Laboratories. A recombinant form of the type-II fibronectin domain of human Factor XII/XIIa (FXII-FNII) was expressed and purified from *E. coli*. A synthetic gene fragment encoding human FXII-FNII was first subcloned into the prokaryotic expression vector pT7HMT (12), and the sequence-confirmed plasmid was used to transform competent cells of *E. coli* strain BL21(DE3). Cells were cultured in 1 L of terrific broth using standard procedures and protein expression was induced overnight at 37°C by addition of IPTG (1 mM final concentration). Unless otherwise indicated, all purification steps were carried out at room temperature. The induced cell pellet was extracted with denaturant, clarified by high-speed centrifugation, and the his-tagged FXII-FNII was purified by NiNTA affinity chromatography according to previously published methods (12). Following elution, the purified protein was reduced with TCEP (1 mM final concentration) at 37°C for 30 min, placed in SpectraPor 3500 dialysis tubing, and dialyzed overnight at room temperature against a buffer of 0.1 M Tris-HCl (pH 8.3), 2.5 M urea, 20 mM glycine, 0.5 mM EDTA, and 2 mM L-cysteine. The next day the sample was dialyzed overnight against 4 L of PBS (pH 7.4). The refolded protein was separated from soluble aggregates by gel-filtration chromatography using a Superdex S75 26/60 column (Cytiva Life Sciences) that had been equilibrated in PBS (pH 7.4). Fractions were analyzed by SDS-PAGE and those containing purified, monomeric FXII-FNII were pooled, concentrated, quantitated, and stored at either 4 or -80°C for later use.

### Crystallization, X-ray Diffraction, Structure Solution, and Refinement

Single crystals of gC1qR-DD were grown by vapor diffusion of hanging drops at 20°C. Briefly, 1 μl of a gC1qR-DD sample (10 mg/ml protein in ddH_2_O) was mixed with an equal volume of a precipitant solution consisting of 0.1 M BisTris-HCl (pH 6.5), 0.1 M sodium chloride, and 1.5 M ammonium sulfate and equilibrated over 500 μl of the same precipitant solution. Plate shaped crystals formed within 2-3 days. Prior to X-ray diffraction analysis, single crystals were cryopreserved by dragging individual samples through a small volume of Santovac Cryo Oil (Hampton Research) and flash cooling in a bath of liquid nitrogen. X-ray diffraction data were collected at 1.000 Å wavelength using beamline 22-BM of the Advanced Photon Source at Argonne National Laboratory. Individual reflections were indexed, integrated, merged, and scaled in the space group *P*6_3_22 using the HKL2000 software package (15).

The structure of gC1qR-DD was solved by molecular replacement using PHASER (16) as implemented in the PHENIX software suite (17) and a monomer of wild-type gC1qR [PDB entry 1P32 (9)] as a search model. Initial electron density maps were calculated at 2.2 Å limiting resolution following placement of a single copy of the gC1qR search model. The structure was then improved by a combination of manual rebuilding using Coot (18) and reciprocal space positional, B-factor, and TLS refinement using PHENIX.REFINE (17). The final polypeptide model consisted of gC1qR residues 76-247, with chain breaks from residues 98-100 and 138-141 due to weak electron density in those areas. In addition, 40 ordered solved molecules were also included in the final model. The refined coordinates and structure factors have been deposited in the PDB using the accession code 7TE3. All representations of protein structures were generated and rendered by PyMol (www.pymol.org/).

### Generation, Purification, and Characterization of anti-gC1qR Monoclonal Antibodies

A sample of wild-type gC1qR was used for the generation of mouse monoclonal antibodies in conjunction with ProMab Biotechnologies. Immunization of experimental mice and preparation of hybridoma lines were carried out according to the proprietary protocols of the vendor. Selection of hybridoma lines for further analysis was based initial quantitation of the anti-gC1qR immunoreactivity present in clonal culture supernatants following direct ELISA against immobilized wild-type gC1qR using standard methods. Generation of ascites fluid in experimental mice and purification of anti-gC1qR mAb IgG using Protein G affinity chromatography was likewise performed using standard procedures by ProMab Biotechnologies. Following purification, the mAbs were buffer exchanged into PBS (pH 7.4), quantitated, aliquoted, and stored at either 4 or -80°C for later use.

Western blotting was used to further characterize anti-gC1qR antibodies. 100 ng of wild-type gC1qR, gC1qR-D1, gC1qR-D2, and gC1qR-DD were separated by Tris-Tricine SDS-PAGE and transferred to a PVDF blotting membrane. Following blocking with a solution of 10% (w/v) non-fat dry milk dissolved in TBS-T (20 mM tris-HCl (pH 8.0), 150 mM NaCl, 0.01% (v/v) Tween-20) for 10 min, each membrane was probed with 1 μg/ml of mAb in blocking solution for 60 min. After a series of washes, each membrane was probed with a 1:10,000 dilution of anti-mouse IgG/IgM HRP conjugate (ThermoFisher #31446) in blocking solution for 30 min. Following further washing, peroxidase activity was detected using SuperSignal West Pico PLUS reagent (ThermoFisher #34580) according to manufacturer’s suggestions.

### Surface Plasmon Resonance Binding Studies

Interactions of various ligands with immobilized forms of gC1qR were investigated by surface plasmon resonance (SPR). All experiments were performed at 25°C on a Biacore T-200 instrument (Cytiva Life Sciences) using a running buffer of HBS-T (20 mM HEPES (pH 7.4), 140 mM NaCl, and 0.005% (v/v) Tween-20) and a flowrate of 30 μl/min. Binding of peptide HKH-20 to wild-type gC1qR and the three gC1qR deletion mutants was initially assessed (1, 19). Samples of various gC1qR proteins were prepared at final concentrations of 10-50 μg/ml in 10 mM acetate buffer (pH 3.5) and coupled to individual flow cells of a CMD200M sensor chip (Xantec Bioanalytics GmbH) using standard amine coupling chemistry. The final immobilization densities achieved were as follows: gC1qR, 361 RU; gC1qR-D1, 487 RU; gC1qR-D2, 360 RU; and gC1qR-DD, 874 RU. A reference surface was also prepared by activation followed by quenching with 1 M ethanolamine (pH 9.5). Solutions of HKH-20 were injected over the biosensor surface in reference subtraction mode using a two-fold series of increasing concentrations ranging from 46.9 nM to 10 µM. Each experimental cycle consisted of an association phase of 1.5 min followed by a dissociation phase of 2 min. Regeneration of the biosensor surface was achieved by an injection of 2 M NaCl for 30 s. Sensorgram series were analyzed using Biacore T-200 Evaluation software v3.2 (Cytiva Life Sciences) by fitting globally to a simple affinity (dose/response) model to derive an apparent K_D_.

Binding of FXII and FXIIa to biotinylated gC1qR and gC1qR-DD was measured next. The general parameters for these experiments were the same to those described above, with the exception that the running buffer contained 50 μM ZnCl_2_ to allow for high-affinity binding of FXII-derived analytes to gC1qR. To begin, gC1qR-avi and gC1qR-DD-avi that had been enzymatically biotinylated were captured to a final level of 100 RU on one and two experimental flow cells of an SA sensor chip, respectively, while a reference surface was prepared by capturing biotin alone. This was followed by injection of a two-fold series of increasing concentrations of FXII and FXIIa ranging from 1.6 to 800 nM across all four flow cells in reference subtraction mode. Each cycle consisted of a 2 min association and 3 min dissociation phase. Regeneration of the biosensor surface was achieved by two sequential injections of 3 mM EDTA for 30 s, which was sufficient to completely dissociate the FXII-derived analytes from captured gC1qR. Sensorgram series were analyzed using Biacore T-200 Evaluation software v3.2 (Cytiva Life Sciences) by fitting globally to a ligand heterogeneity model that accounts for two independent analyte binding sites each described by a set of association and dissociation rate constants.

Binding of monoclonal antibodies to gC1qR was likewise measured by SPR. Wild-type gC1qR that had been chemically biotinylated was captured on experimental flow cells of an SA sensor chip (Cytiva Life Sciences) while a reference flow cell was prepared by capturing biotin alone. Solutions of various mAbs were injected over the biosensor surface in reference subtraction mode using a five-fold series of increasing concentrations ranging from 1.6 to 1000 nM at 30 μl/min. Each experimental cycle consisted of a 2 min association phase at a given mAb concentration, a brief dissociation phase, followed by injection of the next mAb concentration. Once each mAb sample had been injected, a final dissociation phase of 60 min was monitored. Regeneration of the biosensor surface was achieved by three consecutive injections of 0.1 M glycine (pH 2.2), 2 M NaCl for 30 s. Sensorgram series were analyzed using Biacore T-200 Evaluation software v3.2 (Cytiva Life Sciences) by fitting to a bivalent analyte kinetic model to derive the respective association and dissociation rate constants.

Finally, SPR was also used to assess selectivity of mAb-1 for various forms of gC1qR. In this experiment, a solution of mAb-1 was prepared at a final concentration of 10 μg/ml in 10 mM acetate buffer (pH 4.5) and covalently coupled to individual flow cells of a CMD200M sensor chip. An ethanolamine-quenched reference surface was also prepared. Solutions of various gC1qR proteins were injected over the biosensor surface in reference subtraction mode using a five-fold series of increasing concentrations ranging from 0.8 to 500 nM (expressed as gC1qR monomer). Parameters for each experimental cycle, regeneration of the surface, and data analysis were identical to those described in the preceding paragraph.

### Alpha Screen Bead Binding Assays

AlphaScreen bead-based competition binding assays (Perkin Elmer) were used to evaluate the interactions between various anti-gC1qR mAbs and chemically biotinylated wild-type gC1qR. All reaction mixtures consisted of a fixed concentration of anti-gC1qR mAb, a fixed concentration of biotinylated gC1qR (described above), 20 μg/ml anti-mouse AlphaScreen Acceptor beads, 20 μg/ml streptavidin AlphaScreen Donor beads, and a putative competitor molecule. Each reaction was carried out in a final volume of 25 μl using a buffer of 20 mM HEPES (pH 7.4), 140 mM NaCl, 0.1% (w/v) bovine serum albumin, and 0.05% (v/v) Tween-20. The final concentrations for each mAb and biotinylated gC1qR combination were optimized in pair-wise fashion to maximize the dynamic range of the assay according to manufacturer’s suggestions. Step-wise additions of all reagents, incubation times, and data analyses were carried out as previously described (20–22).

### Competition Binding Studies Using Surface Plasmon Resonance

Competition binding studies between various anti-gC1qR mAbs and FXII, FXIIa, and FXII-FNII were carried out by SPR. All parameters for these experiments were the same as those described above where these proteins were used as analytes. To begin, gC1qR-avi that had been biotinylated enzymatically was captured to a final level of either ~100 RU (101 ± 4.4 RU, for n=15 experimental flow cells used in this study) or ~1000 RU (1063 ± 59 RU, for n=15 experimental flow cells used in this study) on all three experimental flow cells of an SA sensor chip, while a reference surface was prepared by capturing biotin alone. Next, to ensure the bioactivity of the captured gC1qR-avi, the second flow cell on each sensor chip was used to perform a single-cycle kinetic study in reference subtraction mode by injecting a five-fold series of increasing mAb concentrations ranging from 1.6 to 1000 nM. Thereafter, a competition study was carried out by presaturating the second flow cell on each sensorchip with 100 nM mAb, followed by injection of a two-fold series of increasing concentrations of FXII-derived analytes across all four flow cells in reference subtraction mode as described above. Each cycle consisted of a 1 min presaturation injection across the second flow cell (carried out at 10 μl/min) prior to a 2 min association and 3 min dissociation phase for each analyte across all four flow cells at 30 μl/min; regeneration of the biosensor surface was achieved by two sequential injections of 3 mM EDTA for 30 s. Sensorgram series were analyzed using Biacore T-200 Evaluation software v3.2 (Cytiva Life Sciences) by fitting globally to a ligand heterogeneity model that accounts for two independent analyte binding sites each described by a set of association and dissociation rate constants. Assessments of precision were obtained by averaging the fitting parameters for the sensorgrams obtained from the third and fourth flow cells of each experiment across five independent biosensor chips at both low and high capture densities. These aggregate values were used as basis for comparison with those obtained following mAb presaturation, which were carried out only a single time for each mAb at low and high capture densities to minimize materials consumption.

## Results

### Generation and Structure/Function Analysis of Loop-Deletion Mutants in gC1qR

Previous structural analyses of the mature form of gC1qR (i.e. residues 74-282) revealed that there are two prominently disordered regions in each gC1qR polypeptide (9). These presumed loop regions connect strands β3-β4 and β5-β6 (9), and are hereafter referred to as “Loop 1” and “Loop 2”, respectively. Both Loop 1 and Loop 2 are rich in acidic amino acids and have been proposed to contribute to the asymmetric charge distribution of the gC1qR trimer (**Figure 1A**). Although the roles of these and other loop regions that connect canonical secondary structure elements in gC1qR have been previously investigated using deletion mutagenesis (23), there exists little detailed information on the structural status of loop-deleted forms of gC1qR. Furthermore, the consequences of deleting these acidic loops simultaneously and/or eliminating their function through loop-targeting monoclonal antibodies remain unexplored.

To address these limitations, we used the existing crystal structure of mature gC1qR to design individual deletion mutants of Loop 1 (i.e. gC1qR-D1) and Loop 2 (i.e. gC1qR-D2) wherein the entire acidic loop was replaced with either two or one glycine residue(s), respectively (**Figure 1A** and **Figure S1**). In addition to this, we designed a double deletion mutant that simultaneously removed both Loop 1 and Loop 2 (i.e. gC1qR-DD) using the same strategy described above (**Figure 1A**). We expressed each form of gC1qR in *E. coli* and purified the mutant proteins for subsequent structure/function analyses (**Figure S1**). Significantly, we found that all three forms of gC1qR were highly soluble and behaved as trimers in solution similarly to wild-type gC1qR (**Figure S1**). To characterize these new mutants in gC1qR, we used a surface plasmon resonance-based approach where forms of gC1qR were covalently coupled through their surface-accessible primary amines to a biosensor flow cell and soluble ligands were injected over each flow cell in reference-correction mode. Although numerous ligands for g1qR have been described (1), we opted to use the human high-molecular weight kininogen (HMWK)-derived peptide, HKH-20 (1, 19), in this initial study since this interaction pair does not require exposure to stringent chemical conditions to regenerate the biosensor surface. As reported previously [**Figure 1B** and (1)], we found that wild-type gC1qR bound HKH-20 with an apparent K_D_ value of 8.7 µM; this corresponded well to the apparent equilibrium dissociation constant we reported for HKH-20 binding to the same protein using an isothermal titration calorimetry approach (6.3 µM) (1). Consistent with the results reported for wild-type gC1qR, we found that both gC1qR-D1 (**Figure 1C**) and gC1qR-D2 (**Figure 1D**) bound to HKH-20 with affinities of ~10 µM. Intriguingly, we also observed a similar affinity for gC1qR-DD binding to HKH-20 (**Figure 1E**). Although this result was somewhat surprising as the HKH-20 peptide is highly positively charged and both loops in gC1qR are rich in negatively charged residues (**Figure 1A**), we interpreted these data to mean that neither acidic loop is directly involved in binding to HKH-20 (1).

**Figure 2:**
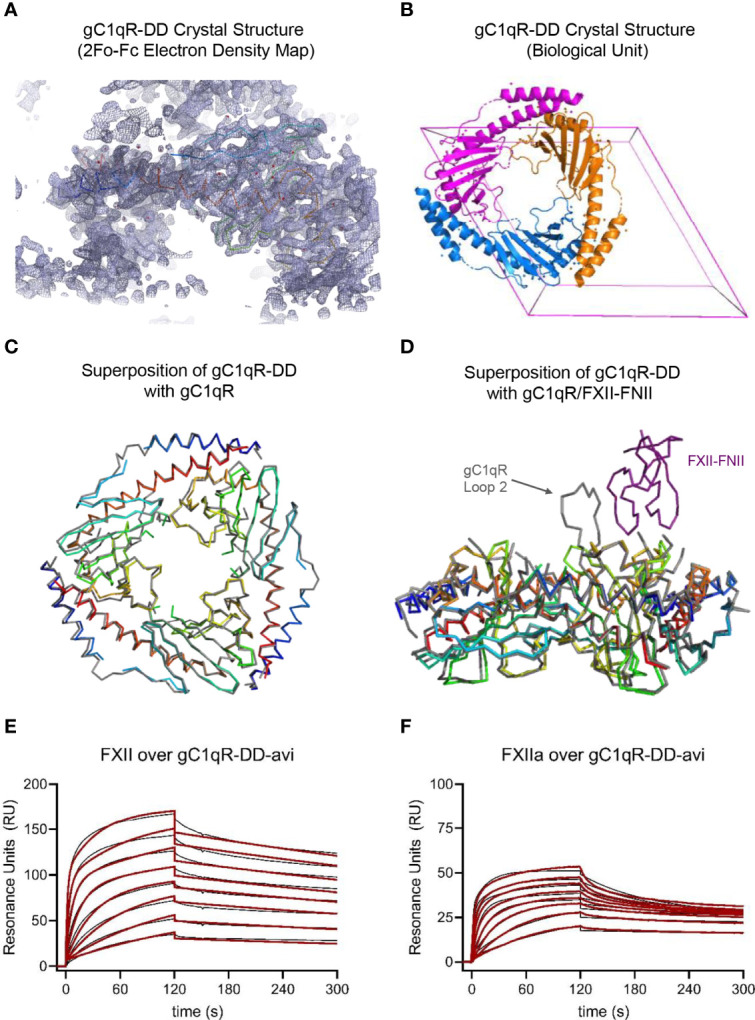
Crystal Structure and Functional Studies of the gC1qR-DD Mutant. **(A)** Correlation of the 2Fo-Fc electron density map contoured at 1.3σ (grey mesh) with the Cα position of the final model (rainbow wire, with N-terminus in blue and C-terminus in red) for the structure of gC1qR-DD determined at 2.2 Å resolution. The U-shaped void at the bottom of this image represents the hole formed upon trimerization of gC1qR-DD monomers. **(B)** Visualization of the gC1qR-DD trimer constructed by applying the crystallographic symmetry operators of the P6_3_22 unit cell (pink box). Individual polypeptides are shown as ribbon diagrams using distinct colors. **(C)** Structural superposition of the gC1qR-DD trimer (rainbow wires) with that of wild-type gC1qR (grey wire). Structures are represented by their Cα positions only for clarity. Coordinates for wild-type gC1qR were obtained from PDB entry 1P32 (9). **(D)** Structural superposition of the gC1qR-DD trimer (rainbow wires) with that of wild-type gC1qR (grey wire) bound to the FNII domain of FXII (purple wire). Structures are represented by their Cα positions only for clarity. Coordinates for wild-type gC1qR bound to FXII-FNII were obtained from PDB entry 6SZW (11). Note that acidic loop 2 contributes a major binding determinant for FXII-FNII (11). **(E)** gC1qR-DD was enzymatically biotinylated through a C-terminal avi-tag and captured to a density of ~100 RU on two experimental flow cells of a streptavidin-derivatized sensor chip. Dose-response sensorgrams for injection of a two-fold dilution series of FXII (800 nM highest concentration) over gC1qR-DD-avi. The black trace represents the experimental data, while the red line represents the outcome of fitting to a ligand heterogeneity kinetic model. **(F)** An identical experiment to that shown in panel **(E)**, with the exception that solutions of FXIIa were used as the analytes (800 nM highest concentration).

To gain further insight into the physical properties of these loop-deleted gC1qR mutants, we crystallized the gC1qR-DD protein. We collected X-ray diffraction data to 2.2 Å limiting resolution and solved the structure of gC1qR-DD by molecular replacement using the published structure of the wild-type gC1qR monomer as a search model (9). Following several rounds of manual rebuilding, our model of gC1qR-DD had R_work_ and R_free_ values of 23.3% and 28.8%, respectively (**Table 1**). The asymmetric unit of the crystal contained a single copy of the gC1qR-DD polypeptide from residues 76-247, aside from two discontinuities in regions connecting α1-β1 (i.e. residues 98-100) and β3-β4 (i.e. residues 138-141) due to weak electron density in those areas. Although the final model contained only a single copy of the gC1qR-DD polypeptide (**Figure 2A**), the structure of the gC1qR-DD trimer that exists in solution could be reconstructed by applying crystallographic symmetry (**Figure 2B**). The structure of the gC1qR-DD trimer superimposes well upon on that of wild-type gC1qR, as 140 of the 165 Cα positions align with a root mean square deviation of 0.544 Å (**Figure 2C**). As expected, the primary areas of structural divergence are found in the areas corresponding to the loop deletions. This feature is particularly striking in a superposition of gC1qR-DD with the structure of gC1qR bound FXII-FNII (**Figure 2D**), wherein Loop 2 adopts a well-ordered conformation that constitutes a major binding determinant for FXII-FNII, and presumably for Factor XII and FXIIa as well (11).

**Table 1:** X-ray Diffraction Data Collection and Refinement Statistics.

	gC1qR-DD (7TE3)
**Data Collection**	
Space group	*P* 6_3_ 2 2
Cell dimensions	
*a*, *b*, *c* (Å)	80.53 80.53 114.55
Resolution (Å)	50.00-2.20 (2.28-2.20)^a^
Wilson B-Factor (Å^2^)	45.4
Completeness (%)	99.9 (99.6)
*I* / σ*I*	22.0 (1.2)
R_pim_	0.032 (0.499)
CC_1/2_	0.996 (0.544)
Redundancy	20.3 (13.8)
**Refinement**	
Resolution (Å)	37.99-2.20
No. reflections	11,768
*R*_work_ / *R*_free_ (%)	23.3 / 28.8
No. atoms	1,367
Protein	1,327
Water	40
Ramachandran Plot	
Favored / Allowed (%)	93.7 / 4.4
B-Factors	
Range & Mean (Å^2^)	29.86-108.12 (55.11)
R.M.S. Deviations	
Bond lengths (Å)	0.007
Bond angles (°)	0.88

a Values in parentheses are for the highest-resolution shell.

Although the FXII-FNII/gC1qR structure highlighted the importance of gC1qR Loop 2 in this interaction (11), both FXII and its activated counterpart, FXIIa, are significantly larger molecules than FXII-FNII (c.f. ~80 kDa to ~10 kDa). Therefore, we wondered whether additional binding sites for FXII and FXIIa might be contributed by the trimeric gC1qR core represented by gC1qR-DD. To examine this possibility, we designed an SPR assay where both wild-type gC1qR and gC1qR-DD that had been biotinylated at a single site *via* a C-terminal avi-tag were captured on separate flow-cells of a streptavidin sensor chip followed by injection of increasing concentrations of FXII or FXIIa. We determined that the interactions between both forms of gC1qR and both forms of FXII required a mechanism incorporating two independent sets of rate constants to adequately describe the experimental data (**Figure S2** and **Table 2**). This feature did not appear to arise from polydispersity in the proteins employed in this study (**Figure S1**). Thus, it likely represented a *bona fide* feature of these interactions that could have been due to the trimeric nature of the gC1qR-avi ligand, conformational changes accompanying FXII binding such as those observed in the FXII-FNII/gC1qR crystal structure described by Kaira et al. (11), or some combination of these factors. In the case of wild-type gC1qR binding to FXII, the first binding site (K_D-1_ = 4.85 ± 1.58 nM) was characterized by a fast on-rate (6.76 ± 1.40x10^5^ M^-1^s^-1^) and slow off-rate (31.6 ± 3.94x10^-4^ s^-1^), while the second binding site (K_D-2_ = 97.6 ± 9.1 nM) had a slower on-rate (2.37 ± 0.24x10^4^ M^-1^s^-1^) but comparable off-rate (2.30 ± 0.02x10^-3^ s^-1^) to the first (**Table 2**). We used a similar strategy to analyze the FXIIa/gC1qR-avi sensorgrams (**Figure S2** and **Table 2**). However, in this case the first binding site was of such high apparent affinity (K_D-1_ = 0.27 ± 0.37 nM) that it approached the limits measurable using the instrumentation available, while the second binding site was somewhat weaker (K_D-2_ = 134 ± 36 nM) than that of FXII. The comparatively large error associated with the first site was due to difficulty in accurately measuring its dissociation rate constant through this approach (**Table 2**). We attempted to address this issue by conducting single-cycle kinetic studies, but the results failed to resolve this problem.

**Table 2:** Interaction Parameters for FXII and FXIIa with Surface Captured gC1qR and gC1qR-DD^a^.

Interaction	k_a-1_ (M^-1^s^-1^)	k_d-1_ (10^-4^ s^-1^)	K_D-1_ (nM)	R_max-1_ (RU)	k_a-2_ (M^-1^s^-1^)	k_d-2_ (10^-3^ s^-1^)	K_D-2_(nM)	R_max-2_ (RU)	χ^2^ (RU^2^)
FXII/ gC1qR-avi^b^	6.76±1.40x10^5^	31.6±3.94	4.85±1.58	47±6	2.37±0.24x10^4^	2.3±0.02	97.6±9.1	104±10	5.5±2.5
FXII/ gC1qR-DD-avi^c^	6.74±0.21x10^5^	11.4±0.43	1.69±0.05	63±12	3.48±0.18x10^4^	0.91±0.13	26.4±4.8	64±9	3.8±1.4
FXIIa/ gC1qR-avi^b^	4.08±0.99x10^5^	0.90±1.2^d^	0.27±0.37^d^	21±1	4.82±0.91x10^4^	6.30±0.51	134±36	48±2	1.4±0.2
FXIIa/ gC1qR-DD-avi^c^	11.3±1.3x10^5^	1.47±1.61^d^	0.12±0.13^d^	24±4	6.37±0.84x10^4^	11.2±2.0	178±35	22±3	0.7±0.2

a Model accounts for two analyte binding sites on the ligand each described by a set of association and dissociation rate constants.

b Values are presented as their mean ± standard deviation obtained from two injections in total for this experiment (one flow cell from two biosensor chips, one replicate). Representative sensorgrams are shown in **Figure S2**.

c Values are presented as their mean ± standard deviation obtained from four injections in total (two flow cells from two biosensor chips, one replicate). Representative sensorgrams are shown in **Figure 2**.

d The large standard deviations associated with these values arise from fitting the dissociation rate constant of the first binding site, which lies near the limit measurable by the instrument.

Intriguingly, we found that surface-captured gC1qR-DD bound with low-nanomolar apparent affinity to both FXII (**Figure 2E**) and FXIIa (**Figure 2F**). For gC1qR-DD binding to FXII, the first FXII binding site was of slightly higher affinity (K_D-1_ = 1.69 ± 0.05 nM) when compared to wild-type gC1qR (**Table 2**). Similarly, the second FXII binding site was also of higher apparent affinity (K_D-2_ = 26.4 ± 4.8 nM) than that of wild-type gC1qR (**Table 2**). Although the first FXIIa binding site of gC1qR-DD followed this trend (K_D-1_ = 0.12 ± 0.13 nM), the second FXIIa binding site was slightly diminished (K_D-2_ = 178 ± 35 nM) when compared to wild-type gC1qR (**Table 2**). In both cases, loss of the acidic loops had a greater influence on the overall saturation levels of the second FXII and FXIIa binding sites of the gC1qR proteins (**Table 2**). This suggested that any contributions of these loops are associated with this lower-affinity FXII/FXIIa binding site. Indeed, we also noticed that loss of the acidic loops resulted in subtle changes to both the shape and the overall response observed throughout all concentrations of FXII and FXIIa injected, even though the global structure/function features of the gC1qR-DD mutant remained intact (**Figures 2E, F**, and **Figure S2**). Thus, while gC1qR-DD clearly bound tighly to both FXII and FXIIa, comparison of this mutant to wild-type gC1qR strongly suggested that interactions of these ligands with gC1qR are mechanistically complex and likely to involve multiple contact sites from each binding partner.

### Generation and Identification of a Loop-Specific Monoclonal Antibody Against gC1qR

Previous reports have described two monoclonal antibodies known as mAb-60.11 and mAb-74.5.2 that are powerful probes of gC1qR structure and function [Reviewed in (1)]. Specifically, whereas mAb-60.11 binds to gC1qR in a manner that interferes with recognition by complement component C1q (10), mAb-74.5.2 binds to gC1qR at site that disrupts its interaction with HMWK (24). Although the epitopes for both mAb-60.11 and mAb-74.5.2 have been mapped at peptide-level resolution on the gC1qR protein (1), neither of these mAbs recognizes residues within or in the vicinity of either of the acidic loop regions in gC1qR. Since these loops appear to play some role in gC1qR binding to certain ligands, notably FXII-FNII [**Figure 2D** and (11)], we sought to develop a novel monoclonal antibody that recognized a loop region within gC1qR and to ascertain its potential as an inhibitor of gC1qR interactions and function.

**Figure 3:**
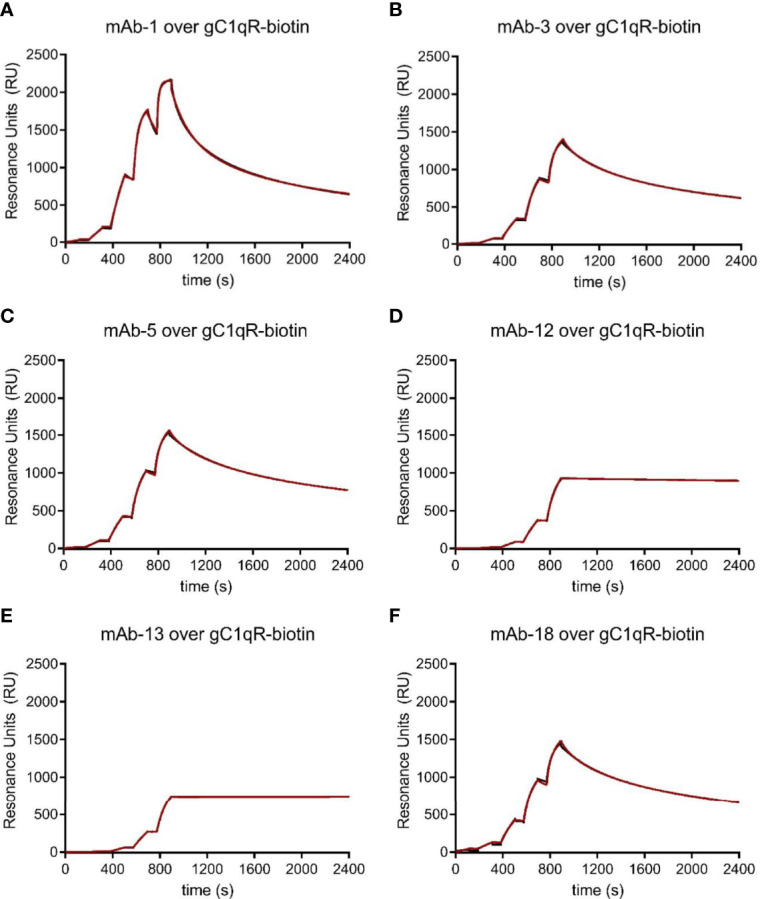
Characterization of a Novel Panel of anti-gC1qR Monoclonal Antibodies. Purified anti-gC1qR monoclonal antibodies were prepared from ascites fluid and their interactions with chemically biotinylated gC1qR were assessed by surface plasmon resonance. **(A)** Reference-corrected sensorgram from a single cycle kinetic study where a five-fold dilution series of mAb-1 (1 μM highest concentration) was injected over a surface of biotinylated gC1qR. The black trace represents the experimental data, while the red line represents the outcome of fitting to a bivalent analyte kinetic model. **(B)** Identical to panel **(A)**, except for mAb-3 served as the analyte. **(C)** Identical to panel **(A)**, except for mAb-5 served as the analyte. **(D)** Identical to panel **(A)**, except for mAb-12 served as the analyte. **(E)** Identical to panel **(A)**, except for mAb-13 served as the analyte. **(F)** Identical to panel **(A)**, except for mAb-18 served as the analyte.

Following immunization of five mice with wild-type gC1qR and generation of B-cell hybridoma cell lines using standard approaches, we screened approximately thirty different lines for those that grew quickly, those that produced relatively high levels of antibody, and those that reacted with gC1qR immobilized on microtiter plates in an ELISA (*Data Not Shown*). We next selected six clones for production of ascites fluid to generate large quantities of antibody and purified the anti-gC1qR IgG using Protein G affinity chromatography. We then characterized each antibody using a series of biochemical assays. Although each antibody selected bound immobilized gC1qR in an ELISA, we first examined the ability of each to bind to immobilized gC1qR in a surface plasmon resonance experiment. Since gC1qR is characterized by an asymmetric charge distribution (9), we again opted against direct coupling of the protein to the biosensor surface; instead, we used a streptavidin surface to capture gC1qR that had been chemically derivatized by a modified biotin group that included a polyethylene glycol-based spacer arm. Using single-cycle kinetic analysis, we determined that each new monoclonal antibody bound with low-nanomolar apparent affinity (<10 nM) to biotinylated gC1qR (**Figure 3**). Examination of experimental data revealed that these antibodies could be clustered into three different groups based upon their sensorgram shapes. mAb-12 and mAb-13 were characterized by the lowest overall signals but clearly had the slowest dissociation rates under these conditions; in addition, these sensorgrams were well-described by Langmuir binding models (*Data Not Shown*), suggesting that only one of the two antigen-binding regions from these intact IgG could interact with its epitope on the captured gC1qR trimer. mAb-3, mAb-5, and mAb-18 had significantly higher overall response levels, but also had faster off-rates under these conditions than either mAb-12 or mAb-13. Finally, mAb-1 had the fastest on-rate and also gave the highest overall response level, suggesting that its epitope was most accessible on the captured gC1qR trimer. Among this collection of antibodies, mAb-1 appeared unique in these properties.

**Figure 4:**
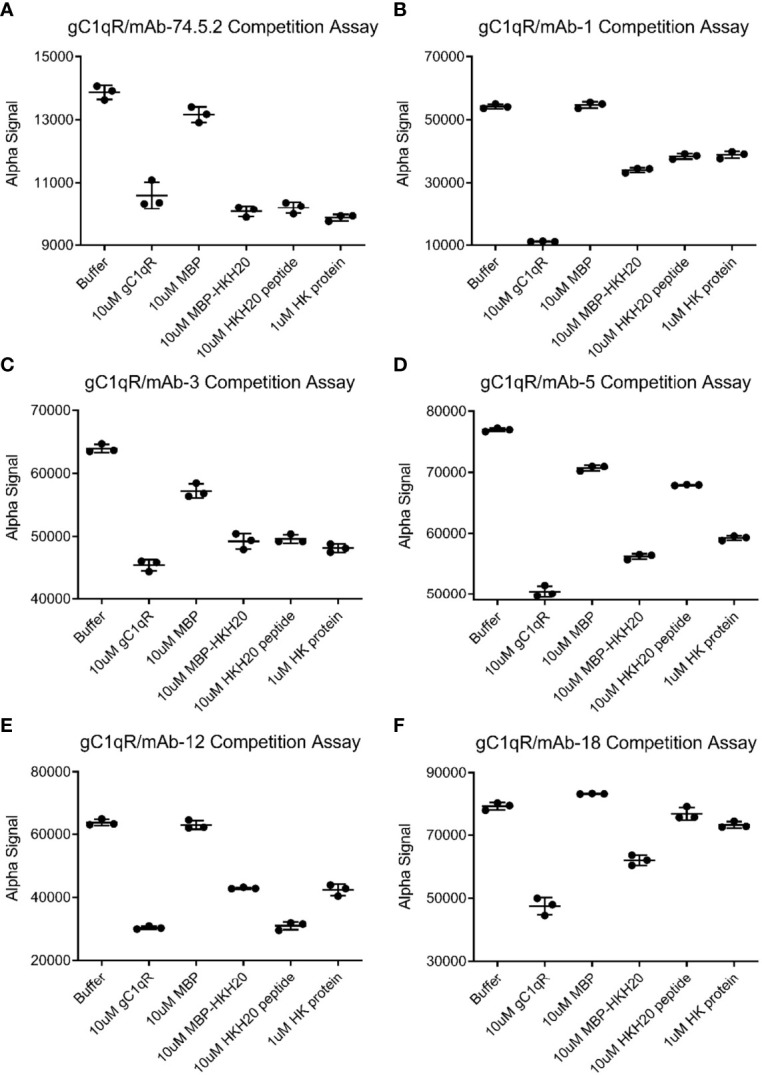
Competitive Behavior of Novel Monoclonal Antibodies on the Interaction of gC1qR with High-Molecular Weight Kininogen (HMWK) Derived Ligands. Various anti-gC1qR monoclonal antibodies were incubated with chemically biotinylated gC1qR and used to generate a luminescent signal using a bead-based AlphaAssay approach. The abilities of a single concentration of HMWK-derived ligands, or various controls, to compete the luminescent signal were measured in triplicate. **(A)** Data for competition of binding between biotinylated gC1qR and mAb-74.5.2, which is known to block binding of HMWK to gC1qR (1, 24). Data shown in this panel were modified from Ghebrehiwet et al. (1). **(B)** Data for competition of binding between biotinylated gC1qR and mAb-1. **(C)** Data for competition of binding between biotinylated gC1qR and mAb-3. **(D)** Data for competition of binding between biotinylated gC1qR and mAb-5. **(E)** Data for competition of binding between biotinylated gC1qR and mAb-12. **(F)** Data for competition of binding between biotinylated gC1qR and mAb-18. Note that the wider black line represents the mean of the three measurements, while the thinner lines are values plus and minus one standard deviation.

The ability of mAb-74.5.2 to compete with HMWK for binding to gC1qR has been documented in both biochemical and functional studies (1, 24). Thus, we next wondered whether any of these newly generated monoclonal antibodies might exhibit similar properties to mAb-74.5.2 and inhibit binding of HMWK to gC1qR. To address this question, we established luminescent bead-based binding assays between each mAb and the chemically biotinylated gC1qR used above (*please see Materials & Methods*) and tested whether this signal could be inhibited by the presence of HMWK itself or defined regions thereof. As expected, we found that the interaction between mAb-74.5.2 and biotinylated gC1qR could be blocked by unlabeled gC1qR (a positive control), as well as purified human HMWK or the HKH-20 peptide [**Figure 4A** and (1)]. We also found that a fusion protein of *E. coli* maltose-binding protein (MBP) and HKH-20 (designed to more accurately mimic the size of HMWK than the HKH-20 peptide alone) inhibited the interaction, whereas MBP itself did not (**Figure 4A**). Interestingly, we found that the signal generated by mAb-1 and biotinylated gC1qR was only diminished by approximately one-half using any of the HMWK ligands described above (**Figure 4B**). mAb-3 exhibited properties that were largely indistinguishable from mAb-74.5.2 (**Figure 4C**), as did mAb-5, with the distinction being that the latter was not as sensitive to the presence of saturating levels of HKH-20 peptide (**Figure 4D**). mAb-12 also behaved somewhat similarly to mAb-74.5.2, with the caveat that its interaction could be fully competed by the HKH-20 peptide but not the HMWK protein (**Figure 4E**); although we did not test it directly, we presume that the same may be true for mAb-13, since its interaction with gC1qR was almost identical to mAb-12 in our previous SPR experiment (**Figures 3D, E**). Finally, we found that mAb-18 binding to biotinylated gC1qR was not effectively competed by HMWK or the HKH-20 peptide (**Figure 4F**). This result was somewhat surprising, as our initial binding studies suggested that mAb-18 might display properties similar to mAb-3 and mAb-5 (**Figures 3B, C, F**). Considering these results, we concluded that several of these monoclonal antibodies recognized epitopes distinct from each other and from that of mAb-74.5.2.

**Figure 5:**
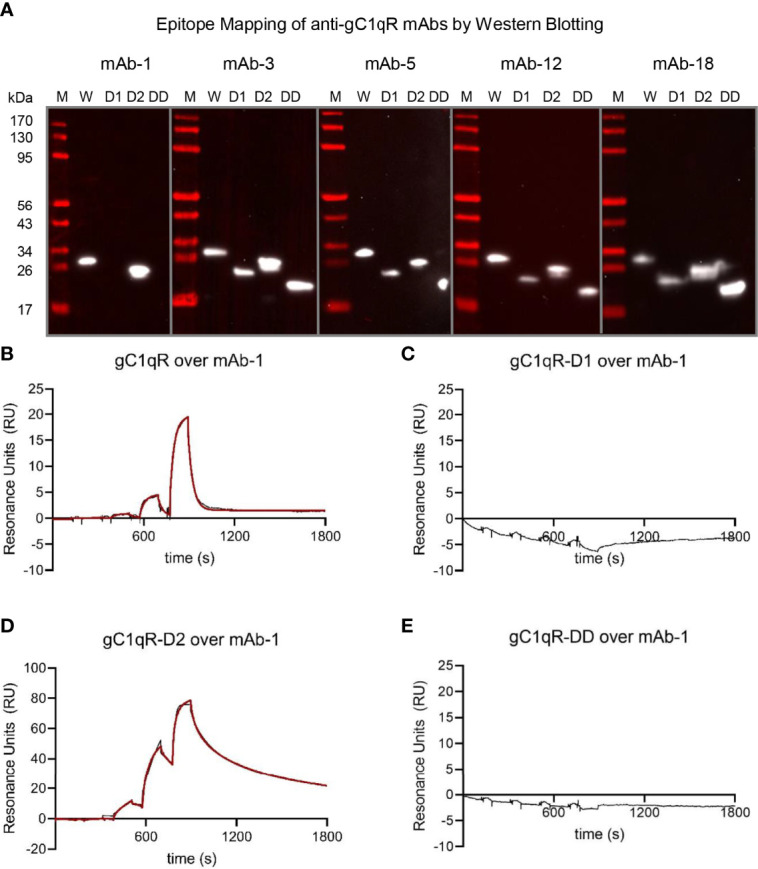
The Epitope of mAb-1 Lies in the First Acidic Loop of gC1qR. The locations of the epitopes for novel anti-gC1qR monoclonal antibodies were mapped using a combination of Western blotting and surface plasmon resonance. **(A)** Epitope mapping by Western blotting. Samples of wild-type gC1qR, gC1qR-D1, gC1qR-D2, and gC1qR-DD were separated by SDS-PAGE, transferred to membranes, and probed with various monoclonal antibodies prior to detection using a chemiluminescent approach. Images representing individual experiments are separated by grey lines, while the values for molecular weight standards are shown at the left of the panel. Note that only mAb-1 showed differential reactivity to forms of gC1qR missing the acidic loops. **(B)** Reference-corrected sensorgram from a single-cycle kinetic study where a five-fold dilution series of wild-type gC1qR (1 μM highest concentration) was injected over a surface of immobilized mAb-1. The black trace represents the experimental data, while the red line represents the outcome of fitting to a kinetic model. **(C)** Identical to panel **(B)**, except that gC1qR-D1 served as the analyte. **(D)** Identical to panel **(B)**, except that gC1qR-D2 served as the analyte. **(E)** Identical to panel **(B)**, except that gC1qR-DD served as the analyte. Note that panels **(C, E)** do not show the outcome of a fit, since there was no appreciable binding of mAb-1 to those analytes.

As final means of characterizing these newly generated monoclonal antibodies, we used our panel of deletion mutants in gC1qR to examine whether any antibody bound to one of the acidic loops. We separated identical quantities of wild-type gC1qR, gC1qR-D1, gC1q-D2, and gC1qR-DD by SDS-PAGE, transferred the samples to a blotting membrane, and processed each membrane for western blotting. Whereas mAb-3, mAb-5, mAb-12, and mAb-18 all reacted equivalently with each form of gC1qR, we found that mAb-1 lost reactivity for both gC1qR-D1 and gC1qR-DD (**Figure 5A**). This result indicated that the epitope recognized by mAb-1 was within the first acidic loop of gC1qR. To investigate this result through an alternative approach, we covalently coupled mAb-1 to a biosensor flow cell and used SPR to measure its ability to bind various forms of gC1qR (**Figures 5B-E**). Consistent with the results obtained by western blotting, we found that wild-type gC1qR (**Figure 5B**) and gC1qR-D2 (**Figure 5D**) bound well to the mAb-1 surface, but neither gC1qR-D1 (**Figure 5C**) nor gC1qR-DD (**Figure 5E**) did so. Interestingly, we noted that gC1qR-D2 appeared to bind more tightly than wild-type gC1qR to mAb-1. This suggested that removal of the second acidic loop may have made the gC1qR epitope recognized by mAb-1 more accessible for interactions. Together, these results identified mAb-1 as a novel monoclonal antibody that recognizes an epitope within the first acidic loop of gC1qR, and provided characterization of several additional anti-gC1qR monoclonal antibodies that could be used for future structure/function studies.

### Multiple Anti-gC1qR Antibodies Interfere With Binding of FXII to gC1qR

When exposed on the endothelial cell surface, gC1qR serves as a scaffold for assembly of a multi-protein complex that leads to activation of the contact system and generation of bradykinin. A component of this complex is FXII and its enzymatically active counterpart, FXIIa. The crystal structure of gC1qR bound to FXII-FNII (11) not only revealed that FXII-FNII binds asymmetrically to the second acidic loop in the gC1qR trimer (**Figure 2D**), it also showed that this loop becomes ordered and forms a zipper motif with a β-strand derived from FXII-FNII (11). Although the acidic loops of gC1qR appear to be intrinsically disordered in the absence of binding partners (9), examination of the unbound gC1qR structure shows that these regions must extend from the same surface of the trimer; this suggests that these loops may be in relatively close physical proximity to one another and, furthermore, that loop specific ligands like mAb-1 might interfere with FXII binding to gC1qR. To test this hypothesis, we designed a competition SPR study. We initially captured gC1qR that had been biotinylated *via* a C-terminal avi-tag on the experimental flow cells of a streptavidin-modified sensorchip. We then compared binding of FXII and FXIIa to a single flow cell that was presaturated with an anti-gC1qR antibody prior to each injection series with two control flow cells that were not (**Figure S3**).

**Figure 6:**
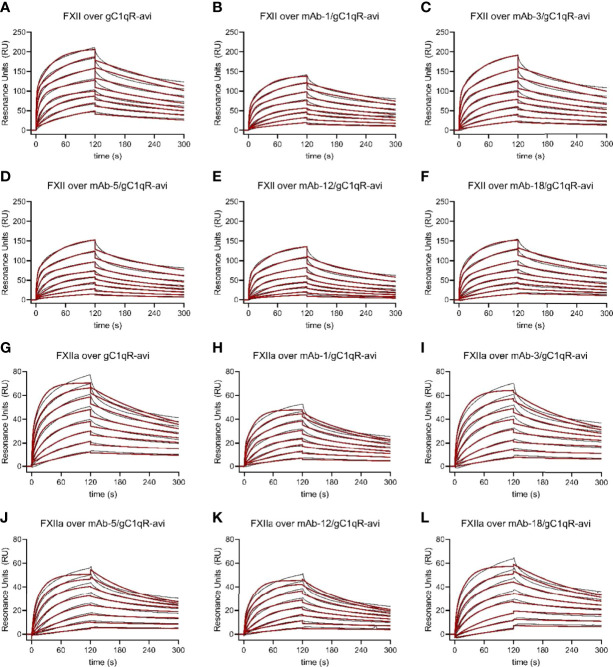
Multiple anti-gC1qR Antibodies Interfere with Binding of Factor XII and Factor XIIa to gC1qR. Wild-type gC1qR was enzymatically biotinylated through a C-terminal avi-tag and captured to a density of ~100 RU on the experimental flow cells of a streptavidin-derivatized sensor chip. After confirmation of mAb binding to the captured gC1qR-avi using a single-cycle kinetic experiment (*Data Not Shown*), the impact of that mAb on FXII and FXIIa binding to gC1qR was assessed by pre-loading a single flow cell with saturating levels of the mAb followed by injecting various concentrations of FXII and FXIIa over all three experimental flow cells. **(A)** Dose-response sensorgram series (black traces) and fits (red traces) for injection of FXII over a gC1qR-avi surface. **(B)** Analogous to panel **(A)**, except with presaturation of mAb-1. **(C)** Analogous to panel **(A)**, except with presaturation of mAb-3. **(D)** Analogous to panel **(A)**, except with presaturation of mAb-5. **(E)** Analogous to panel (A), except with presaturation of mAb-12. **(F)** Analogous to panel **(A)**, except with presaturation of mAb-18. **(G)** Dose-response sensorgram series (black traces) and fits (red traces) for injection of FXIIa over a gC1qR-avi surface. **(H)** Analogous to panel **(G)**, except with presaturation of mAb-1. **(I)** Analogous to panel **(G)**, except with presaturation of mAb-3. **(J)** Analogous to panel **(G)**, except with presaturation of mAb-5. **(K)** Analogous to panel **(G)**, except with presaturation of mAb-12. **(L)** Analogous to panel **(G)**, except with presaturation of mAb-18.

We utilized five independent sensorchips in this study at a low gC1qR capture density and five at a high gC1qR capture density. We were able to reproducibly capture ~100 RU of gC1qR-avi on each experimental flow cell of the first cohort and ~1000 RU on each experimental flow cell of the second (*please see Materials & Methods*). Consequently, we obtained multiple independent measurements of FXII and FXIIa binding to gC1qR-avi (n=10) at each density, which allowed a detailed assessment of data reproducibility across this entire set of experiments (**Figure S3** and **Table S1**). Although both FXII and FXIIa bound strongly to the gC1qR-avi surface as before, we again found the sensorgrams fit poorly to a Langmuir binding model. However, by again invoking a mechanism with a second set of rate constants, we found that the FXII/gC1qR-avi interaction was well-described by two independent binding sites (**Figure 6A** and **Table S1** c.f. **Table 2**).

We then compared the parameters for FXII and FXIIa binding to captured gC1qR-avi to gC1qR-avi that had been presaturated with various monoclonal antibodies (**Figure 6**, **Figures S3**, **S4**, and **Table S1**). As we hypothesized from examination of the FXII-FNII/gC1qR structure (11) and the epitope mapping study described above (**Figure 5**), we found that presaturation with mAb-1 diminished the signal from injecting either FXII (**Figure 6B**) or FXIIa (**Figure 6H**) by roughly 40% across all concentrations when compared to controls. Inspection of the fitting parameters suggested that perturbation of both binding sites was responsible for this effect, as the overall saturation level (i.e. R_max-1_ and R_max-2_) dropped to a comparable extent when compared to the control values (**Table S1**). We also found that mAb-1 partially inhibited FXII and FXIIa binding to gC1qR-avi at higher gC1qR capture densities (**Figure S4** and **Table S1**). Interestingly, we found that mAb-3 was a poor inhibitor of FXII (**Figure 6C**) and FXIIa (**Figure 6I**) binding at low gC1qR-avi capture densities, but comparable to mAb-1 at higher gC1qR-avi capture densities (**Figure S4**). Surprisingly, when we presaturated the gC1qR-avi surface with either mAb-5 (**Figure 6D**), mAb-12 (**Figure 6E**), or mAb-18 (**Figure 6F**), we observed generally similar inhibition of FXII binding as we found for mAb-1 (**Figure 6A**), and the same was also true for binding of FXIIa (**Figures 6J–L**). Moreover, we observed a similar effect on FXII and FXIIa binding at higher gC1qR capture densities following presaturation with mAb-1, mAb-5, mAb-12, and mAb-18 (**Figure S4**).

Examination of the fitting parameters from experiments on both FXII and FXIIa failed to identify any single antibody or group of antibodies that were more potent inhibitors than the others (**Figure S3** and **Table S1**), even though these antibodies have diverse biochemical properties and appear to recognize distinct epitopes on gC1qR (**Figures 3**–**5**). Furthermore, while select antibodies (i.e. mAb-3) manifest increased potency as a function of gC1qR capture densities (**Figures 6** and **S4**), the resulting inhibitory effect never exceeded that displayed by any of the others. As a group, the most obvious effect these antibodies had on gC1qR binding to FXII and FXIIa was partial lowering of both apparent R_max_ values when compared to uninhibited controls (**Figure S3** and **Table S1**). Although multiple anti-gC1qR antibodies interfere with binding of FXII and FXIIa to gC1qR to an extent, no antibody in this collection was entirely effective in blocking these interactions completely.

## Discussion

gC1qR at the surface of endothelial cells can bind to C1q, whereby it triggers the classical pathway of complement, as well as FXII, HMWK, and PK, whereby it initiates both the contact system of coagulation and the kinin-generating system (1). Despite the fact that they are conceptually distinct, these pathways appear to participate in extensive crosstalk with one another in a variety of physiological and pathophysiological settings (25, 26). While further defining these interrelationships is a topic of active investigation, the large number of gC1qR ligands (1) and the paucity of detailed structural and biochemical information on how these interactions form remains a significant obstacle to therapeutic development. A major breakthrough in this area was made recently when Kaira et al. reported the structure of the FXII-FNII bound to gC1qR (11). Although FXII-FNII represents only a small fragment (~10 kDa) of the much larger FXII protein (~80 kDa including glycosylation), this structure revealed important details about FXII/gC1qR interactions. Among these were the identification of the second acidic loop of gC1qR (i.e. residues 190-201) as a key binding determinant for asymmetric assembly of the gC1qR/FXII and gC1qR/FXII/HMWK complexes (11). Whereas the binding site for FXII-FNII is present in each gC1qR polypeptide, only one site appears to be occupied in the context of the gC1qR trimer. Steric occlusion of the otherwise equivalent binding sites has been suggested to be responsible for preventing additional FXII-FNII from binding to gC1qR (11). Presumably, this effect would be exacerbated in the case of full-length FXII, or in the presence of other large ligands such as HMWK.

Since both acidic loops project from the same face of the gC1qR trimer (9), we hypothesized that large molecules, including antibodies, that bind to either of the acidic loops of gC1qR might inhibit FXII binding. In this study, we identified a new monoclonal antibody (mAb-1) that bound specifically to an epitope within the first acidic loop of gC1qR (i.e. residues 139-163) (**Figure 5**); although these residues do not represent the FXII-FNII binding site *per se*, the apparent physical proximity of the acidic loops to one another in the gC1qR structure (9) suggested that this antibody might still interfere with FXII binding. Indeed, we found that presaturation with mAb-1 reduced the number of available binding sites, as judged by its effects on the R_max_ associated with both binding sites on gC1qR for FXII (**Figures 6**, **S4** and **Table S1**). Although we observed a similar effect for mAb-1 on binding of FXIIa (**Figures 6**, **S4**, and **Table S1**), we found that inhibition of FXII-FNII binding appeared to be slightly more modest (**Figure S5**). We suspect that the presence of the ~150 kDa antibody bound to acidic loop 1 could not fully block access of the much smaller FXII-FNII (~10 kDa) to its binding site on acidic loop 2. Curiously, though, we found that several other monoclonal antibodies which recognize distinct gC1qR epitopes from mAb-1 (**Figures 3**–**5**) also seemed to inhibit FXII binding to a similar extent (**Figures 6**, **S3**, **S4**, and **Table S1**). We interpret this to mean that the presence of almost any exogenous gC1qR ligand the size of a ~150 kDa antibody might inhibit binding of the ~80 kDa FXII to gC1qR to some degree. We believe this concept might be generally relevant to this system, since all the monoclonal antibodies examined here except mAb-18 also perturbed binding of the ~110 kDa HMWK to gC1qR (**Figure 4**). While we only investigated their effects on binary gC1qR complexes, the ability of our monoclonal antibodies to interfere with the higher-order gC1qR/FXII/HMWK assemblies identified by Kaira et al. (11) seems likely.

Although they recognize distinct epitopes on gC1qR (**Figures 3**–**5**), we found that each of the monoclonal antibodies described here binds gC1qR with a relatively high apparent affinity (**Figure 3**). This raises questions as to why each these monoclonal antibodies appear to block FXII binding partly, but not completely (**Figures 6**, **S3**, **S4**, and **Table S1**). Paradoxically, we believe that size is an important consideration in this respect as well. Examination of the gC1qR structure shows that its ring-like assembly is approximately 75 Å in diameter (9). Examination of the structure for an intact IgG-class antibody however reveals that the distal ends of its two antigen-binding F(ab) motifs are separated by greater than 140 Å through space (27). While the hinge regions of antibodies clearly provide for some flexibility, it seems unlikely that both antigen-binding sites of an intact monoclonal antibody could bind their cognate epitopes in a single gC1qR trimer. Consistent with this idea, we noted that the sensorgrams obtained by injecting either mAb-12 or mAb-13 over gC1qR were well-described by Langmuir binding models (*Data Not Shown*). In light of these considerations, we believe the trimeric assembly of gC1qR presents a unique quandary for inhibitor design such that intact monoclonal antibodies may not be the best route forward. Although intact antibodies may be able to bridge adjacent gC1qR trimers such as those proposed by Kaira et al. (11), smaller fragments of high affinity monoclonal antibodies, such as F(ab) or scFv, or alternatives such as camelid-derived nanobodies (i.e. VHH), may provide high-affinity binders in a smaller overall size that could more effectively occupy each of the potential binding sites in a single gC1qR trimer. Such molecules might also provide the advantage of not activating downstream immune effector functions against endothelia, including the classical complement pathway and/or antibody-dependent cytotoxicity *via* FcγR-bearing immune cells, as this might have undesirable consequences in diseases like hereditary angioedema. Clearly, additional work will be needed to identify inhibitors with these properties and to fully evaluate them in appropriate models of disease.

Whereas the acidic loop regions of gC1qR have received considerable attention in our work here and elsewhere (9–11), the most prominent structural feature of the gC1qR trimer is its ring-like assembly (9, 10). In the present report, we provided additional information on this aspect of gC1qR by determining a 2.2 Å resolution crystal structure of a gC1qR double loop deletion mutant (i.e. gC1qR-DD). We found that gC1qR-DD behaved as a trimer in solution and this arrangement was likewise visible in its crystal structure (**Figure 2**). We also found that gC1qR-DD bound tightly to both FXII and FXIIa with apparent affinity in the low nanomolar range (**Figure 2** and **Table 2**). This latter result was particularly intriguing considering the FXII-FNII/gC1qR crystal structure (11), which could be interpreted as arguing that the second acidic loop is the only determinant of gC1qR binding to FXII and FXIIa. Yet considering the FXII-FNII/gC1qR structure (11) alongside the information presented here (**Figure 2** and **Table 2**), we suspect that the interaction between FXII/FXIIa and gC1qR most likely involves sites derived from the second acidic loop of gC1qR as well as its trimeric core and, equally importantly, additional regions of FXII besides its N-terminal FNII domain. Indeed, such a model is easiest to reconcile with the extensive binding studies we present here (**Figures 2**, **6**, **Table 2**, **Figures S2**, **S4**, and **Table S1**). Although additional structural information would be invaluable in deciphering how gC1qR binds FXII/FXIIa, obtaining it is likely to be challenging since this interaction is not adequately described by reductionist biochemical models (e.g. domain fragments) even though these are often more tractable for structural studies.

There remain some inconsistencies in the literature regarding how gC1qR interacts with HMWK. However, both our group (1) and others (11) have shown previously that peptides derived from HMWK residues 493-516 bind gC1qR with affinities in the 1-10 µM range independently of metal-ion cofactor. Here we expanded on this observation by showing that none of our loop deletion mutants in gC1qR lost their ability to bind the HMWK-derived peptide HKH-20 in an SPR study (**Figure 1**). Again, these results were consistent with that those of Kaira et al., who used a combination of deletion mutagenesis and isothermal titration calorimetry to show that a region encompassing residues 214-224 of gC1qR is a critical determinant for HMWK binding (11). It is interesting to note that these residues lie at the bottom of the central cavity formed upon gC1qR trimerization (9) and that this area is not perturbed in the gC1qR-DD crystal structure (**Figures 2C, D**). Still, this raises questions about not only these residues’ accessibility for HMWK binding, but whether any structural rearrangements need to occur in gC1qR prior to binding HMWK. We believe continued dissection of these questions from a structurally-oriented perspective will be required not only to understand how gC1qR, HMWK, and FXII interact with one another, but to determine how best to block these interactions for therapeutic purposes.

## Data Availability Statement

The datasets presented in this study can be found in online repositories. The names of the repository/repositories and accession number(s) can be found below: PDB, accession code: 7TE3.

## Author Contributions

YZ: designed and performed protein-protein interaction experiments and analyzed data. AV: assisted with characterization of monoclonal antibodies and binding studies. EK: assisted with characterization of monoclonal antibodies and binding studies. XX: assisted with characterization of monoclonal antibodies and binding studies. NP: assisted with characterization of monoclonal antibodies and binding studies. KR: assisted with characterization of monoclonal antibodies, binding studies, and X-ray crystallography. BGa: assisted with characterization of monoclonal antibodies, binding studies, and analyzed data BGh: designed of the overall scope of the study and wrote the manuscript. BGe: designed of the overall scope of the study, analyzed data, and wrote the manuscript. All authors contributed to the article and approved the submitted version.

## Funding

This research was supported by U.S. National Institutes of Health grant R35GM140852 to BGe and grant R56AI122376 to BGh. We also acknowledge the Terry C. Johnson Center of Kansas State University for its generous support of the initial stages of this work and for support of AV and EK as Cancer Research Awardees.

## Conflict of Interest

The authors declare that the research was conducted in the absence of any commercial or financial relationships that could be construed as a potential conflict of interest.

## Publisher’s Note

All claims expressed in this article are solely those of the authors and do not necessarily represent those of their affiliated organizations, or those of the publisher, the editors and the reviewers. Any product that may be evaluated in this article, or claim that may be made by its manufacturer, is not guaranteed or endorsed by the publisher.
