# Supplementary material for: gC1qR/C1qBP/HABP-1: Structural Analysis of the Trimeric Core Region, Interactions With a Novel Panel of Monoclonal Antibodies, and Their Influence on Binding to FXII
Source: Front Immunol. 2022 Jul 5;13:887742. doi: 10.3389/fimmu.2022.887742 (PMC9294231; doi:10.3389/fimmu.2022.887742)
Supplement: Supplementary file 1 [file DataSheet_1.pdf]

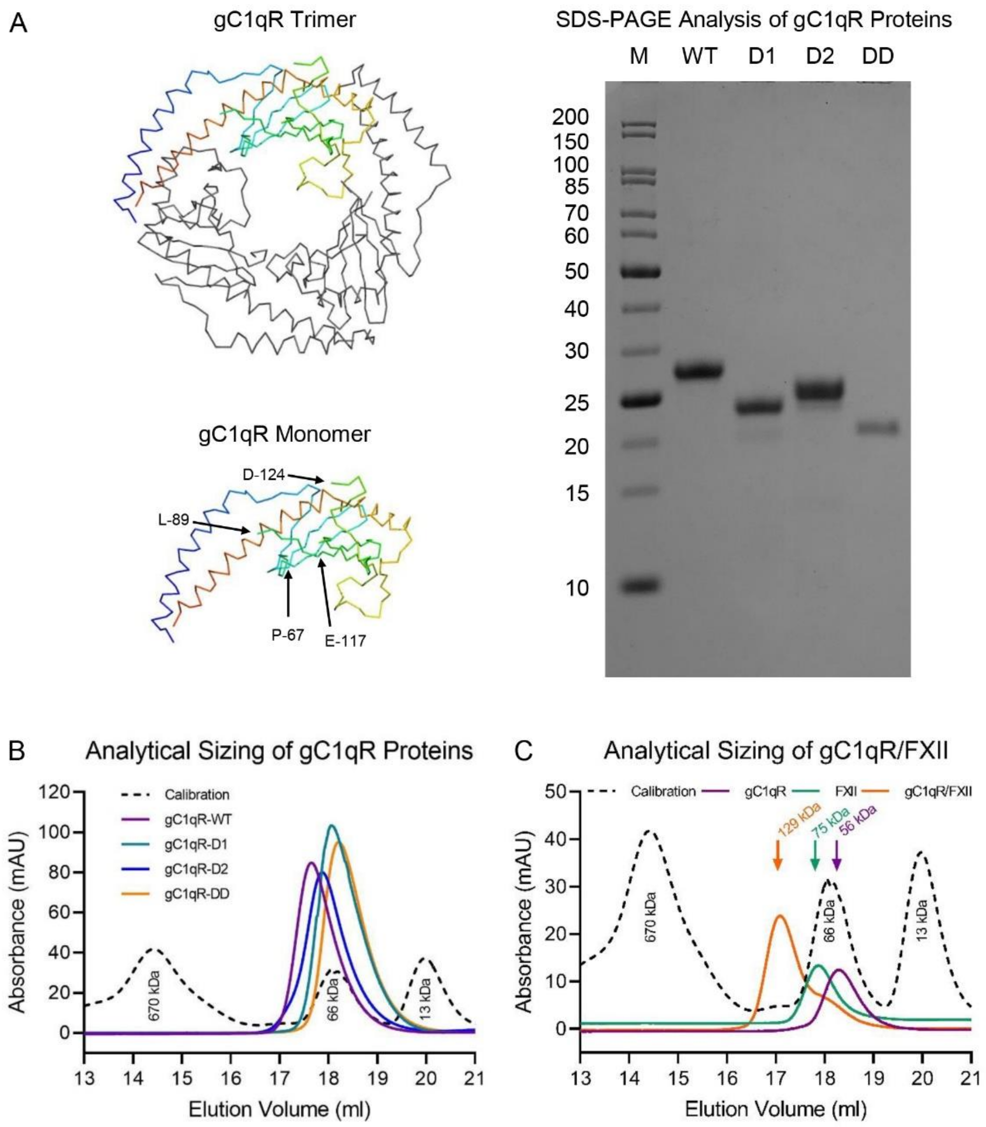

Figure S1. Physical Characterization of gC1qR Proteins and Complexes Employed in this Study. (A) Structure of the wild-type gC1qR protein and design of loop deletion mutants therein. The structure of the gC1qR trimer drawn from PDB entry 1P32 is shown atop the left panel with an individual monomer colored with its N-terminus in blue and its C-terminus in red. The structure of the same monomer is shown the lower left panel, with the locations of key residues marking the boundaries of the loop regions indicated by arrows; the numbering of these residues corresponds to Figure 1A of the manuscript. SDS-PAGE analysis of purified gC1qR proteins is shown in the right panel. Samples of purified gC1qR proteins were prepared under reducing conditions and separated by 10% Tris-Tricine SDS-PAGE prior to staining with Coomassie Brilliant Blue. M, molecular weight markers (New England Biolabs #P7717); WT, wild-type gC1qR; D1, loop 1 deletion mutant; D2, loop 2 deletion mutant; DD, double loop deletion mutant. (B) Analytical gel filtration chromatography of purified gC1qR proteins. Samples of gC1qR proteins shown in panel A were separated on a Superdex 200 Increase 10/300 GL column and compared to the size standards thyroglobulin (670 kDa), serum albumin (66 kDa), and a single V-set Ig domain protein (13 kDa). Fitting to the calibration curve yielded apparent molecular weights of 87, 65, 74, and 59 kDa, respectively. This is consistent with a trimeric solution structure for each gC1qR protein studied here. (C) Analytical gel filtration chromatography of gC1qR/FXII complexes using an analogous strategy to panel B. Samples of purified FXII, wild-type gC1qR in a Zn^{++}-containing buffer, and gC1qR/FXII in a Zn^{++}-containing buffer, were separated and compared to size standards. Fitting to the calibration curve yielded an apparent molecular weight of 129 kDa for the gC1qR/FXII complex, which corresponds to a gC1qR trimer binding to a FXII monomer under these conditions.

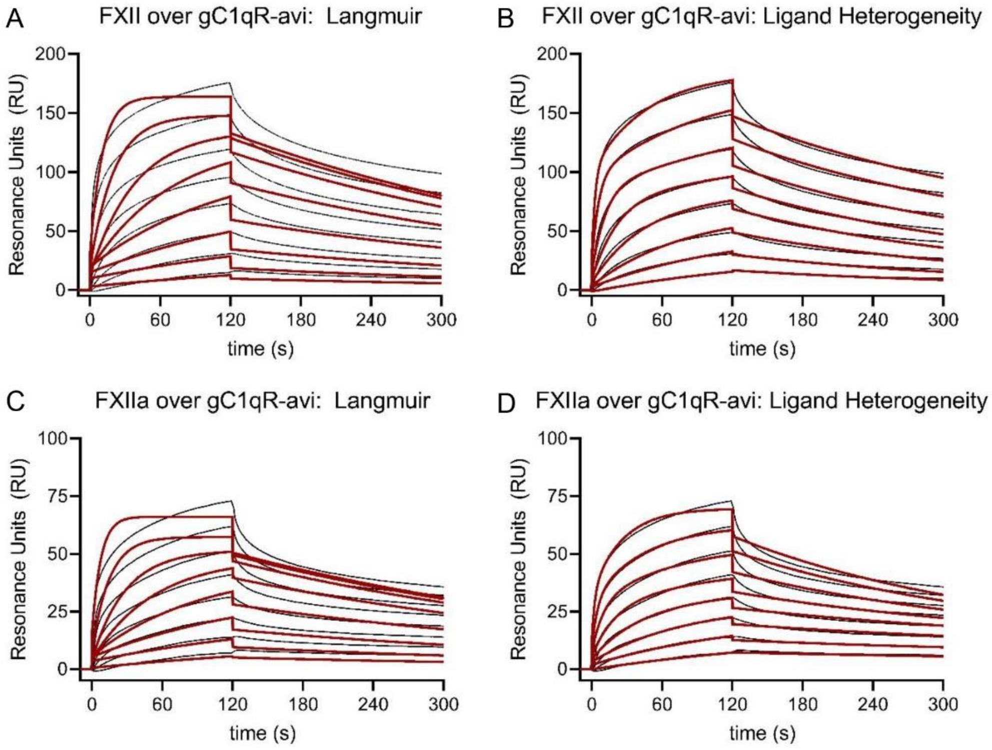

Figure S2. Comparision of FXII and FXIIa Binding to Low-Density gC1qR-avi Surfaces. Biotinylated gC1qR-avi was captured on all three experimental flow-cells of a streptavidin-coated sensor chip at a level of ~100 RU. A two-fold concentration series ranging from 6.25 nM-800 nM of either FXII (panels A, B) or FXIIa (panels C, D) was then injected at a flow-rate of 30 μl/min. The reference corrected sensorgram series were fit to either Langmuir (panels A, C) or Ligand Heterogeneity (panels B, D) kinetic models. The mean χ^2 values across three experimental flow cells were as follows: FXII/Langmuir, 52.7; FXII/Heterogenity, 4.2; FXIIa/Langmuir, 7.4; FXIIa/Heterogeneity, 1.1. A simliar trend was observed in binding studies using high-density gC1qR-avi surfaces with a capture level of ~1000 RU (*Data Not Shown*).

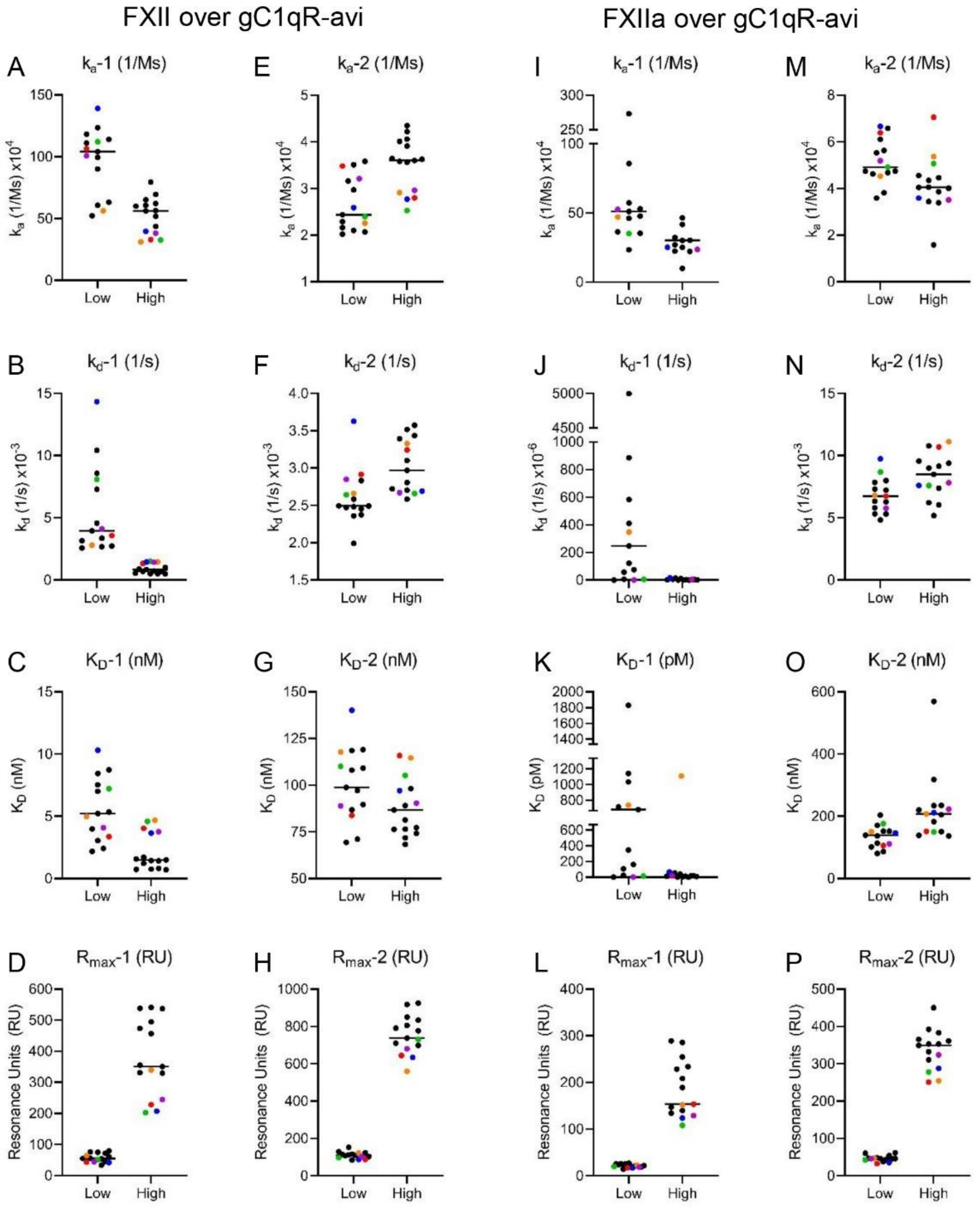

Figure S3. Comparison of Fitting Parameters for Binding of FXII and FXIIa to gC1qR Using Either Low or High-Density Surfaces. Reference corrected sensorgram series were obtained following injection of FXII and FXIIa over surfaces of enzymatically biotinylated gC1qR-avi that had been captured at either low-density (~100 RU) or high-density (~1,000 RU). Data were fit globally to a kinetic model that incorporates two independent analyte binding sites described by independent sets of rate constants. The individual values (n=10) in the absence of competitor were plotted as filled circles based upon the surface density employed. Values obtained in the presence of saturating levels of mAbs are colored as follows: mAb-1, red; mAb-3, orange; mAb-5, green; mAb-12, blue; mAb-18, purple. Aside from the expected differences in R_{max} for each site which are dependent on surface density, there is good agreement between the control observations made at low and high density. The exception to this seems to be the first FXIIa binding site on gC1qR, whose apparent affinity approaches the limit reliably measured by this instrument; these errors are exacerbated at high surface densities of gC1qR, most likely due to mass-transport effects. (A-D) Parameters associated with the first FXII binding site on gC1qR. (E-H) Parameters associated with the second FXII binding site on gC1qR. (I-L) Parameters associated with the first FXIIa binding site on gC1qR. (M-P) Parameters associated with the second FXIIa binding site on gC1qR.

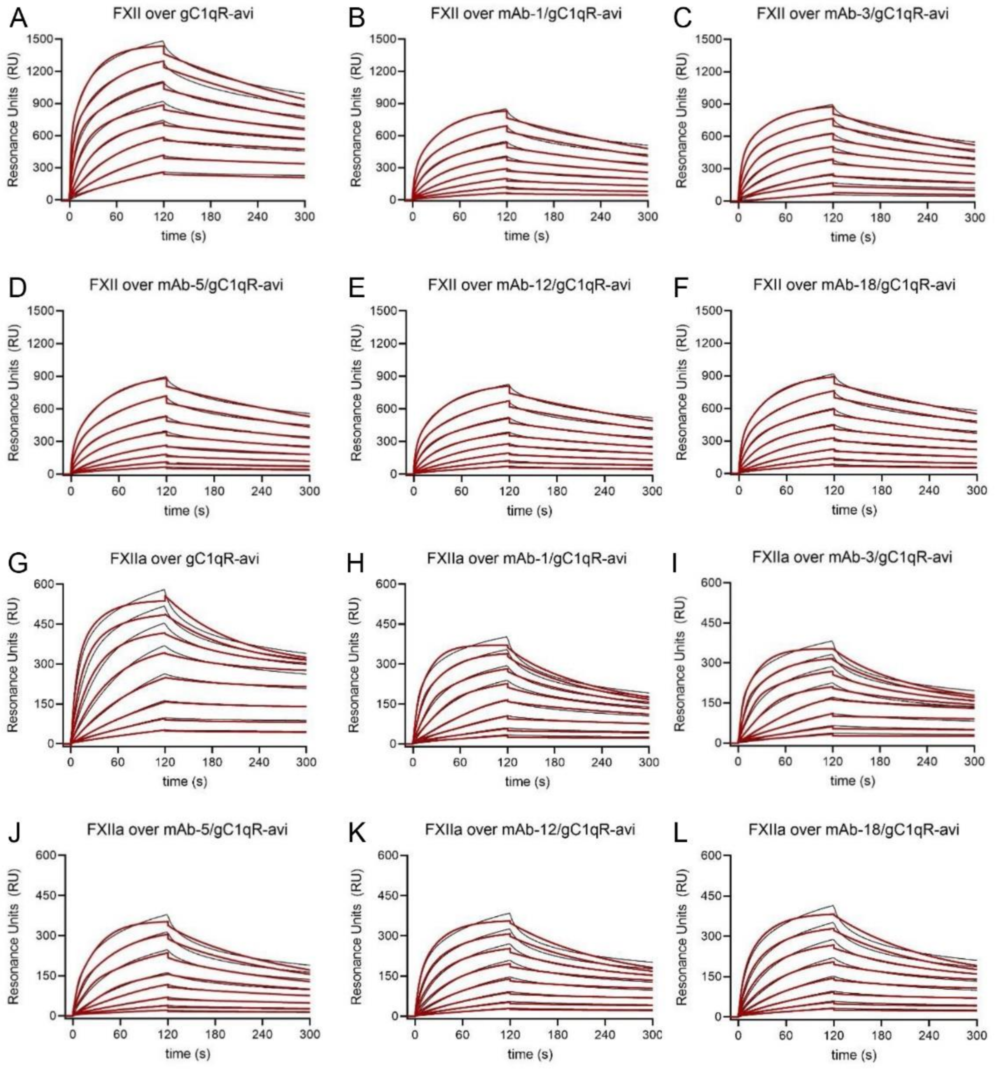

Figure S4. Multiple anti-gC1qR Antibodies Interfere with Binding of Factor XII and Factor XIIa to gC1qR. Wild-type gC1qR was enzymatically biotinylated through a C-terminal avi-tag and captured to a density of ~1000 RU on the experimental flow cells of a streptavidin-derivatized sensor chip. After confirmation of mAb binding to the captured gC1qR-avi using a single-cycle kinetic experiment (*Data Not Shown*), the impact of that mAb on FXII or FXIIa binding to gC1qR was assessed by pre-loading a single flow cell with saturating levels of the mAb followed by injecting various concentrations of FXII or FXIIa over all three experimental flow cells. (A) Dose-response sensorgram series (black traces) and fits (red traces) for injection of FXII over a gC1qR-avi surface. (B) Analogous to panel A, except with presaturation of mAb-1. (C) Analogous to panel A, except with presaturation of mAb-3. (D) Analogous to panel A, except with presaturation of mAb-5. (E) Analogous to panel A, except with presaturation of mAb-12. (F) Analogous to panel A, except with presaturation of mAb-18. (G) Dose-response sensorgram series (black traces) and fits (red traces) for injection of FXIIa over a gC1qR-avi surface. (H) Analogous to panel G, except with presaturation of mAb-1. (I) Analogous to panel G, except with presaturation of mAb-3. (J) Analogous to panel G, except with presaturation of mAb-5. (K) Analogous to panel G, except with presaturation of mAb-12. (L) Analogous to panel G, with presaturation of mAb-18.

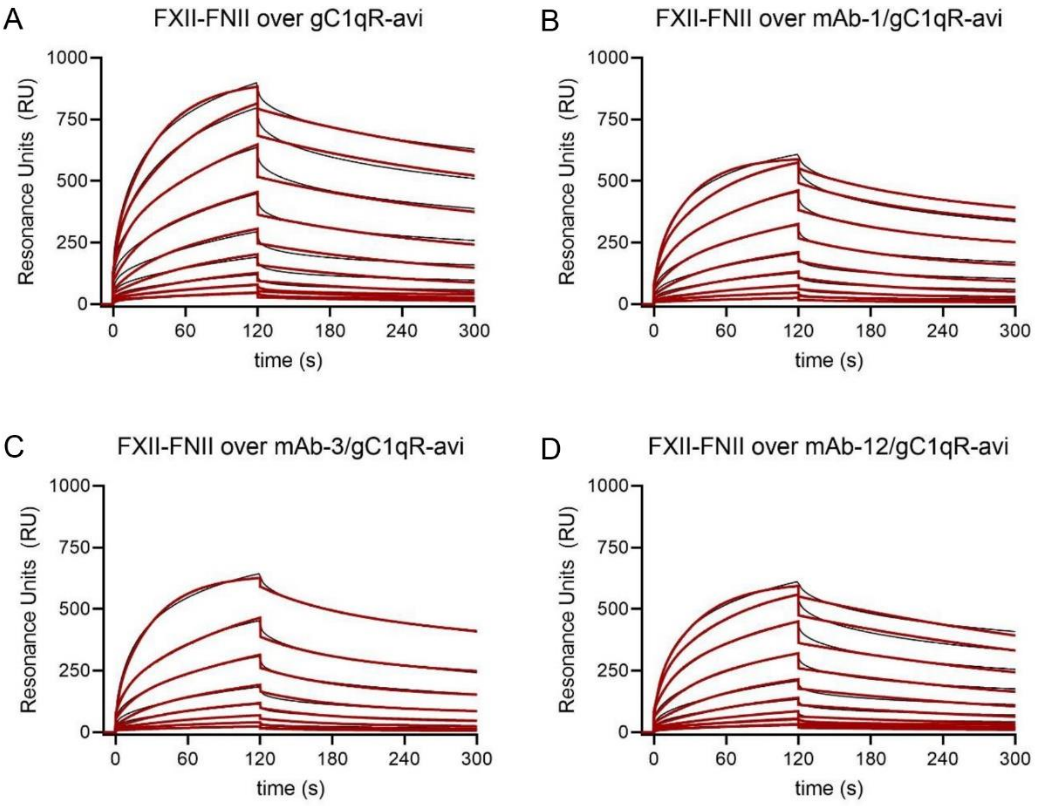

E **Interaction Parameters for FXII-FNII with Immobilized gC1qR-avi[a]**

Analyte	Inhibitor	$k_{a\text{-}1}$ ($M^{-1}s^{-1}$)	$k_{d\text{-}1}$ (10^{-4} s^{-1})	$K_{D\text{-}1}$ (nM)	$R_{max\text{-}1}$ (RU)	$k_{a\text{-}2}$ ($M^{-1}s^{-1}$)	$k_{d\text{-}2}$ (10^{-3} s^{-1})	$K_{D\text{-}2}$ (nM)	$R_{max\text{-}2}$ (RU)
FXII-FNII	-	269±23.4	4.94±1.10	1820±275	680±39.5	5870±2120	11.4±4.07	1960±355	131±31.2
	mAb-1	330	6.83	2070	443	3370	11.8	3510	129
	mAb-3	302	9.27	3070	505	3730	16.8	4490	118
	mAb-12	266	12.2	4600	450	2690	4.59	1710	139

[a]Model accounts for two analyte binding sites on the ligand each described by a set of association and dissociation rate constants.
[b]Values for interactions assessed in the absence of mAb presaturation are presented as their mean ± standard deviation obtained from eight replicate injections in total (two flow cells from four biosensor chips using a high capture density).

Figure S5. Multiple anti-gC1qR Antibodies Interfere with Binding of the FNII Domain of Factor XII to gC1qR. Wild-type gC1qR was enzymatically biotinylated through a C-terminal avi-tag and captured to a density of ~1000 RU on the experimental flow cells of a streptavidin-derivatized sensor chip. After confirmation of mAb binding to the captured gC1qR-avi using a single-cycle kinetic experiment (*Data Not Shown*), the impact of that mAb on binding of FXII-FNII to gC1qR was assessed by pre-loading a single flow cell with saturating levels of the mAb followed by injecting various concentrations of FXII-FNII over all three experimental flow cells. (A) Dose-response sensorgram series for injection of FXII-FNII over a gC1qR-avi surface. (B) Analogous to panel A, except with presaturation of mAb-1. (C) Analogous to panel A, except with presaturation of mAb-3. (D) Analogous to panel A, except with presaturation of mAb-12. (E) Table containing the fit parameters for the experimental series shown in panels A-D.

Table S1. Interaction Parameters for FXII and FXIIa with gC1qR in the Presence of anti-gC1qR mAbs[a]

Interaction	Inhibitor	k_{a-1} ($M^{-1}s^{-1}$)	k_{d-1} (10^{-4} s^{-1})	K_{D-1} (nM)	R_{max-1} (RU)	k_{a-2} ($M^{-1}s^{-1}$)	k_{d-2} (10^{-3} s^{-1})	K_{D-2} (nM)	R_{max-2} (RU)
FXII/ gC1qR-avi[b] (Low Dens)	-	9.37x10^5	49.4	5.39	61	2.63x10^4	2.45	96.8	115
	mAb-1	10.6x10^5	35.8	3.37	43	3.48x10^4	2.92	83.8	88
	mAb-3	5.64x10^5	28.1	4.98	64	2.26x10^4	2.66	118	122
	mAb-5	11.2x10^5	81.0	7.22	51	2.40x10^4	2.64	110	97
	mAb-12	13.9x10^5	143	10.3	42	2.59x10^4	3.63	140	88
	mAb-18	10.1x10^5	41.2	4.08	44	3.21x10^4	2.85	88.9	102
FXIIa/ gC1qR-avi[b] (Low Dens)	-	7.09x10^5	7.38	0.60	22	5.01x10^4	6.42	134	49
	mAb-1	34.2x10^5	80.2	2.35	17	6.39x10^4	6.74	106	33
	mAb-3	4.71x10^5	3.48	0.74	23	4.53x10^4	6.79	150	47
	mAb-5	3.51x10^5	0.05	0.01	20	4.92x10^4	8.68	176	42
	mAb-12	32.7x10^5	70.3	2.15	17	6.66x10^4	9.75	147	35
	mAb-18	5.25x10^5	0.01	0.002	19	5.19x10^4	5.77	111	46
FXII/ gC1qR-avi[c] (High Dens)	-	6.05x10^5	6.92	1.19	441	3.86x10^4	3.08	80.0	805
	mAb-1	3.30x10^5	13.3	4.03	228	2.80x10^4	3.24	116	645
	mAb-3	3.12x10^5	14.6	4.68	340	2.91x10^4	3.33	115	560
	mAb-5	3.27x10^5	15.0	4.59	203	2.53x10^4	2.66	105	731
	mAb-12	3.97x10^5	14.6	3.66	207	2.77x10^4	2.69	97	635
	mAb-18	3.82x10^5	14.3	3.75	245	2.96x10^4	2.67	90	680
FXIIa/ gC1qR-avi[c] (High Dens)	-	2.87x10^5	0.04	0.02	211	3.78x10^4	8.12	239	365
	mAb-1	18.7x10^5	55.0	2.94	153	7.06x10^4	10.7	151	251
	mAb-3	15.4x10^5	17.1	1.11	152	5.37x10^4	11.1	207	254
	mAb-5	23.1x10^5	96.7	4.18	108	5.07x10^4	7.59	150	278
	mAb-12	2.52x10^5	0.17	0.07	124	3.59x10^4	7.60	212	288
	mAb-18	2.36x10^5	0.05	0.02	129	3.51x10^4	7.82	223	324

[a]Model accounts for two analyte binding sites on the ligand each described by a set of association and dissociation rate constants.
[b]Control values (no inhibitor) for low density surfaces are presented as their mean from ten injections in total (two flow cells from five biosensor chips, one replicate). Representative sensorgrams are shown in Fig. 6. Estimates of precision across all injections can be obtained from Fig. S3.
[c]Control values (no inhibitor) for high density surfaces are presented as their mean from ten injections in total (two flow cells from five biosensor chips, one replicate). Representative sensorgrams are shown in Fig. S4. Estimates of precision across all injections can be obtained from Fig. S3.
